# Inflammation Drives Phosphorylation and Acetylation of MutS Homolog 3 and Interaction with Cytosolic HDAC6

**DOI:** 10.7150/jca.131728

**Published:** 2026-03-25

**Authors:** Stephanie S. Tseng-Rogenski, Minoru Koi, John M. Carethers

**Affiliations:** 1 Department of Internal Medicine, University of Michigan, Ann Arbor, MI, USA.; 2 Division of Gastroenterology and Hepatology, Department of Medicine, University of California San Diego, San Diego, CA, USA.; 3 Moores Cancer Center, University of California San Diego, San Diego, CA, USA.; 4 Herbert Wertheim School of Public Health and Longevity Science, University of California San Diego, San Diego, CA, USA.

**Keywords:** MSH3, MutSβ, mismatch repair, HDAC6, colorectal cancer, interleukin-6, microsatellite instability, acetylation, phosphorylation, post-translational modification

## Abstract

**Background:**

MutS Homolog 3 (MSH3), part of the MutSβ DNA mismatch repair complex with MSH2, can reversibly translocate from the nucleus to cytosol via IL-6 signaling, abrogating nuclear MutSβ function and is associated with metastasis and poor patient survival. A polymorphism consisting of deletion of 27-bp proximate to the nuclear localization signal (NLS) (Δ27bpMSH3) allows MSH3 cytosolic retention with IL-6 or oxidative stress. Here, we examined for IL-6-induced post-translational modifications associated with MSH3 cytosolic translocation.

**Methods:**

We utilized MSH3-genotyped colon cancer cell lines after IL-6 treatment to assess post-translational modification of MSH3 via Western blots (WB). We modified sequences within the MSH3-NLS-EGFP reporter construct to assess MSH3 localization via immunofluorescent microscopy and WB after nuclear-cytosolic fractionation. Immunoprecipitation (IP) followed by WB was used to study post-IL-6-induced interactions with MSH3.

**Results:**

MSH3 and Δ27bpMSH3 increased serine phosphorylation after 2 hours followed by tyrosine phosphorylation 18 hours post IL-6 treatment, with Δ27bpMSH3 showing more robust phosphorylation than MSH3 likely due to increased cytosolic translocation. MSH3 cytosolic localization was enhanced by acetylation of lysine residues within MSH3's NLS, specifically at residues K^99^, K^100^ and K^103^. With the observed acetylation control for MSH3 cytosolic localization, IP experiments demonstrate binding of cytosolic-located histone deacetylase 6 (HDAC6) to acetylated Δ27bpMSH3.

**Conclusions:**

Polymorphic MSH3 undergoes serine/tyrosine phosphorylation and NLS acetylation upon IL-6 signaling for its nuclear-cytosolic shift and binds HDAC6 in the cytosol which may contribute to anticipated deacetylation and MSH3 protein stability when separated from MSH2. These modifications might be targeted to regulate MSH3's intracellular localization.

## Introduction

Human DNA mismatch repair (MMR) is constituted by two recognition complexes, MutSα (heterodimer of MSH2 and MSH6) and MutSβ (heterodimer of MSH2 and MSH3), followed by the common signaling complex MutLα (heterodimer of MLH1 and PMS2) to maintain DNA post-synthetic replication fidelity prior to mitosis in cells 1-5. MutSα is able to recognize and bind single base mispairs and mono- and up to di-nucleotide microsatellite insertion-deletion loops (IDLs), as well as altered nucleotides such as O^6^-methylguanine and 5-fluorodeoxyuracil 2-10. MutSβ recognizes dinucleotide and longer microsatellite IDLs as its principle biochemical function and can partially recognize 5-fluorodeoxyuracil 9,11. There are two inherited cancer-prone syndromes driven by germline mutation of MMR genes. Lynch syndrome, comprising 3% of all CRC patients, demonstrate germline mutation of *MSH2*, *MLH1*, *MSH6*, or *PMS2* (and *EPCAM*) followed by somatic inactivation of the second allele in tissue. The extremely rare Constitutional Mismatch Repair Deficiency Syndrome demonstrates biallelic germline MMR gene mutations in patients, principally that of *PMS2* and *MSH6* 4,12. There are also three sporadic conditions caused by non-germline defects in MMR. Sporadic microsatellite instability-high (MSI-H) colorectal cancers (CRCs), observed in 15% of all CRC patients, arise from epigenetic biallelic hypermethylation of *MLH1* 1-5. Lynch-like syndrome, observed in 1% of all CRC patients, is driven by biallelic somatic mutations in *MSH2*, *MLH1*, *MSH6*, or *PMS2* 4,13. Finally, elevated microsatellite alterations at selected tetranucleotide repeats (EMAST) are observed in 50% of CRCs and is caused by loss-of-function of MSH3 protein and is associated with metastatic colorectal cancer and poor patient survival 14-17. Thus, of all the conditions caused by defects in MMR, MSH3 dysfunction is the most prevalent among CRC patients. Additionally, biallelic germline *MSH3* mutations have been observed in four families world-wide, and affected patients showcase complete loss of MSH3 expression within normal and tumor tissue and exhibit EMAST, with a syndrome tumor profile consisting of adenomatous oligopolyposis, CRCs, breast, endometrial, gastric and lung cancer, and astrocytoma 18,19. Germline monoallelic mutation of *MSH3* has not been shown to be a contributory cause for Lynch syndrome 4,19.

The somatic loss-of-function mechanism of MSH3 in 50% of CRCs occurs via pro-inflammatory IL6/JAK/STAT3 signaling and/or oxidative stress to trigger a reversible nuclear-to-cytosolic shift of MSH3, removing it from the nucleus and the site of DNA repair when in the cytosol 14,20-23. The nucleus-cytosolic protein shift is unique to MSH3, as other MMR proteins do not translocate under IL-6 signaling 21. MSH3 has two nuclear export signals that cooperate with each other for MSH3's movement out of the nucleus, and a single nuclear localization signal (NLS) for initial entry after translation and re-entry back into the nucleus that is encoded for in exon 1 of *MSH3* that also possesses six repeats of an imperfect 9-bp tandem repeat (G[C]CCG[C]CAGCC) representing a polyalanine and proline-rich component ([A]_12_PPAPPAPA) proximate to the NLS 22. *MSH3* exon 1 polymorphisms occur in and around the polyalanine and proline-rich component that can alter NLS function. A specific exon 1 polymorphism caused by an imperfect deletion of 27 base pairs, *Δ27bpMSH3*, modifies MSH3's protein structure such that the NLS is less accessible to engage with nuclear pore proteins for nuclear import 23. Consequently, Δ27bpMSH3 becomes sequestered in the cytosol after IL-6 signaling or oxidative stress or DNA doubles strand breaks, as compared to the reversibility for wild type MSH3 protein 20-22,24.

Because IL-6 signaling induces nuclear-cytosolic translocation of MSH3, we examined MSH3 for post-translational changes upon IL-6 signaling. We observed both phosphorylation and acetylation of MSH3 with IL-6 signaling as post-translational modifications that may allow MSH3 to bind to HDAC6.

## Results

### MSH3 becomes serine then tyrosine phosphorylated after IL-6 treatment

**Figure 1:**
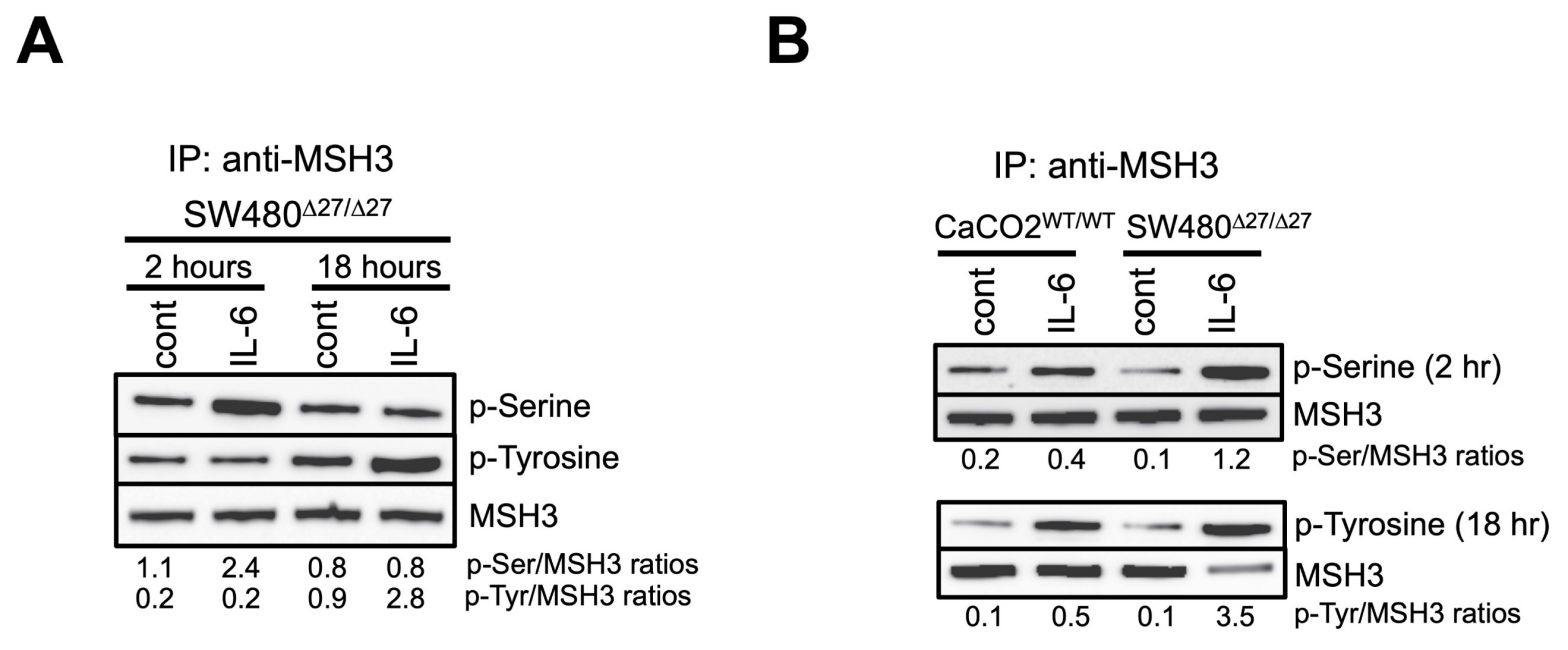
** MSH3 and Δ27bpMSH3 increases serine then tyrosine phosphorylation after IL-6 treatment.** After IL-6 treatment, cell whole lysates were utilized for immunoprecipitation (IP) and Western blotting. (***A***) MSH3 immunoprecipitation experiments from SW480^Δ27/Δ27^ cell protein demonstrated phosphorylation on serine residue(s) 2 hours after IL-6 treatment and demonstrated phosphorylation on tyrosine residue(s) 18 hours after treatment. (***B***) MSH3 immunoprecipitation experiments using CaCO2^WT/WT^ and SW480^Δ27/Δ27^ cell protein demonstrating serine and tyrosine phosphorylation. Thus, both MSH3 isoforms follow the same serine then tyrosine phosphorylation pattern after IL-6 treatment, with phosphorylation of Δ27bpMSH3 more robust than MSH3. p=phosphor, Ser=serine, Tyr=tyrosine, cont=control, WT=wild type.

Our prior observation of IL-6-induced MSH3 nuclear-to-cytosolic translocation suggests post-translational modifications may be involved in intracellular translocation. We previously observed with IL-6 treatment that the MSH3 nuclear to cytosolic shift is detected as early as 2.5 hours after treatment, peaks between 8 to 16 hours, and subsides 24 hours after treatment 21. To directly examine if MSH3 underwent phosphorylation upon IL-6 treatment, we performed immunoprecipitation (IP) experiments using a polyclonal anti-MSH3 antiserum, followed by Western blotting (WB) using anti-phospho-Serine (p-Ser), anti-phospho-Tyrosine (p-Tyr), and anti-phospho-Threonine (p-Thr) antibodies. We first utilized protein from SW480^Δ27/Δ27^ cells which are genotyped biallelic for Δ27bpMSH3 and for which we previously showed readily translocated to the cytosol upon IL-6 signaling and becomes sequestered in that compartment 22. IL-6 caused Δ27bpMSH3 to increase serine phosphorylation from baseline 2 hours after treatment which dissipated by 18 hours post-IL-6 (**Figure 1A**). Subsequently and metachronous to serine phosphorylation, Δ27bpMSH3 increased tyrosine phosphorylation from baseline by 18 hours post-IL-6 treatment (**Figure 1A**). To assess for any differences in level and timing of phosphorylation between Δ27bpMSH3 and MSH3, we again performed IP for MSH3 followed by WB for phosphorylation with CaCo2^WT/WT^ cells, genotyped biallelic for WT MSH3 22. As shown in **Figure 1B**, both Δ27bpMSH3 and MSH3 follow the pattern of serine phosphorylation by 2 hours and lagged tyrosine phosphorylation by 18 hours post IL-6 treatment. The robustness of serine and tyrosine phosphorylation was more prominent with Δ27bpMSH3 likely because it exhibited higher level of IL-6 facilitated cytosol location compared to MSH3. We did not observe detectable signals for threonine phosphorylation for either Δ27bpMSH3 or MSH3 (*data not shown*). The serine phosphorylation coincides with our prior observation of just before the MSH3 nuclear-to-cytosolic shift, while the tyrosine phosphorylation occurs well after the shift.

### MSH3 is acetylated after IL-6 treatment, with nuclear-cytosolic translocation regulated by acetylation of three lysine residues within its nuclear localization signal (NLS)

**Figure 2:**
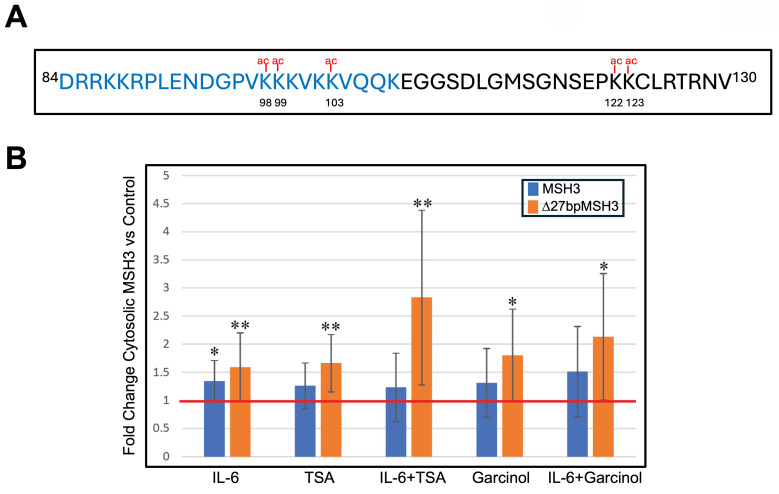
** Acetylation of MSH3 exon 1 lysine residues affect MSH3 and Δ27bpMSH3 cytosolic localization.** (***A***) MSH3 exon 1 protein sequence showing position of five lysine residues predicted to be acetylated, with three of the lysine residues directly within the nuclear localization signal (NLS) sequence (blue letters). (***B***) CaCO2^WT/WT^ and SW480^Δ27/Δ27^ cells were treated with IL-6, trichostatin A (TSA, a deacetylation inhibitor), and Garcinol (a histone acetyltransferase inhibitor), then underwent nuclear-cytosolic fractionation and Western blotting. Note that IL-6 increased both cytosolic MSH3 and Δ27bpMSH3 whereas TSA and Garcinol more selectively increased cytosolic Δ27bpMSH3. All treatments led to increased percentage of cytosolic Δ27bpMSH3 [(IL-6 vs. control, *P* = 0.0092); (TSA vs. control, *P* = 0.0042), (IL-6 & TSA vs. control, *P* = 0.0079); (Garcinol vs. control, *P* = 0.0298); (IL-6 & Garcinol vs. control, *P* = 0.0247)], while only IL-6 treatment resulted in higher fraction of cytosolic MSH3 (*P* = 0.0171). Overall, Δ27bpMSH3 over MSH3 is more easily affected by acetylation of exon 1 lysine residues to aid nuclear exit and cytosolic accumulation. Red line = normalized control level.

We previously mapped MSH3's NLS to near the N terminus of the protein and encoded within exon 1 of *MSH3* 22. MSH3's NLS is a bipartite signal spanning from codon 84 to codon 114 (^84^DRRKKRPLENDGPVKKKVKKVQQKEGGSDLG^114^) with the second cluster of K/R residues more critical than the first cluster of K/R residues for its nuclear import function 22. Five lysine residues of MSH3 have been reported to be acetylated 21, with three lysine residues (K^98^, K^99^, K^103^) being within the NLS and two lysine residues (K^122^, K^123^) adjacent to the NLS (**Figure 2A**). Because acetylation is the most common post-translational modification in eukaryotic cells 26, we investigated if acetylation on these lysine residues could impact MSH3 shuttling between the cytosol and nucleus. We performed nuclear-cytosolic fractionation followed by Western blotting on protein from SW480^Δ27/Δ27^ and CaCo2^WT/WT^ cells to assess acetylation and cytosolic location of both Δ27bpMSH3 and MSH3. IL-6 treatment, as we have previously demonstrated, increased the cytosolic fraction of both Δ27bpMSH3 and MSH3 (**Figure 2B**). To assess the effect of acetylation has on MSH3's cytosolic location, we treated cells with Trichostatin A (TSA), a de-acetylation inhibitor, and Garcinol, a histone acetyltransferase inhibitor. TSA is expected to prevent MSH3 de-acetylation while Garcinol might prevent MSH3 acetylation if it is a target of Garcinol. Surprisingly, both treatments appeared to increase cytosolic MSH3 (**Figure 2B**). We then treated cells with the combination of IL-6 and TSA or Garcinol, which appeared to cause synergistic increased cytosolic fraction of Δ27bpMSH3 although not significantly different than single treatment (e.g. IL-6/TSA vs. IL-6, *P* = 0.0592; IL-6/Garcinol vs. TSA, *P* = 0.0598), and no significant increase for that of MSH3 (**Figure 2B**). These findings are consistent with IL-6 facilitated cytosolic transfer and retention of Δ27bpMSH3 through protein acetylation. We note that MSH3 did increase its cytosolic fraction with IL-6 and with both inhibitors, but only IL-6 treatment reached statistical significance for MSH3 increased cytosolic fraction. Additionally, none of the inhibitor or IL-6 treatments affected distributions of other MMR proteins (**Supplementary Figure 1**). Overall, our data indicates that the acetylation status of Δ27bpMSH3 is associated with its subcellular localization.

**Figure 3:**
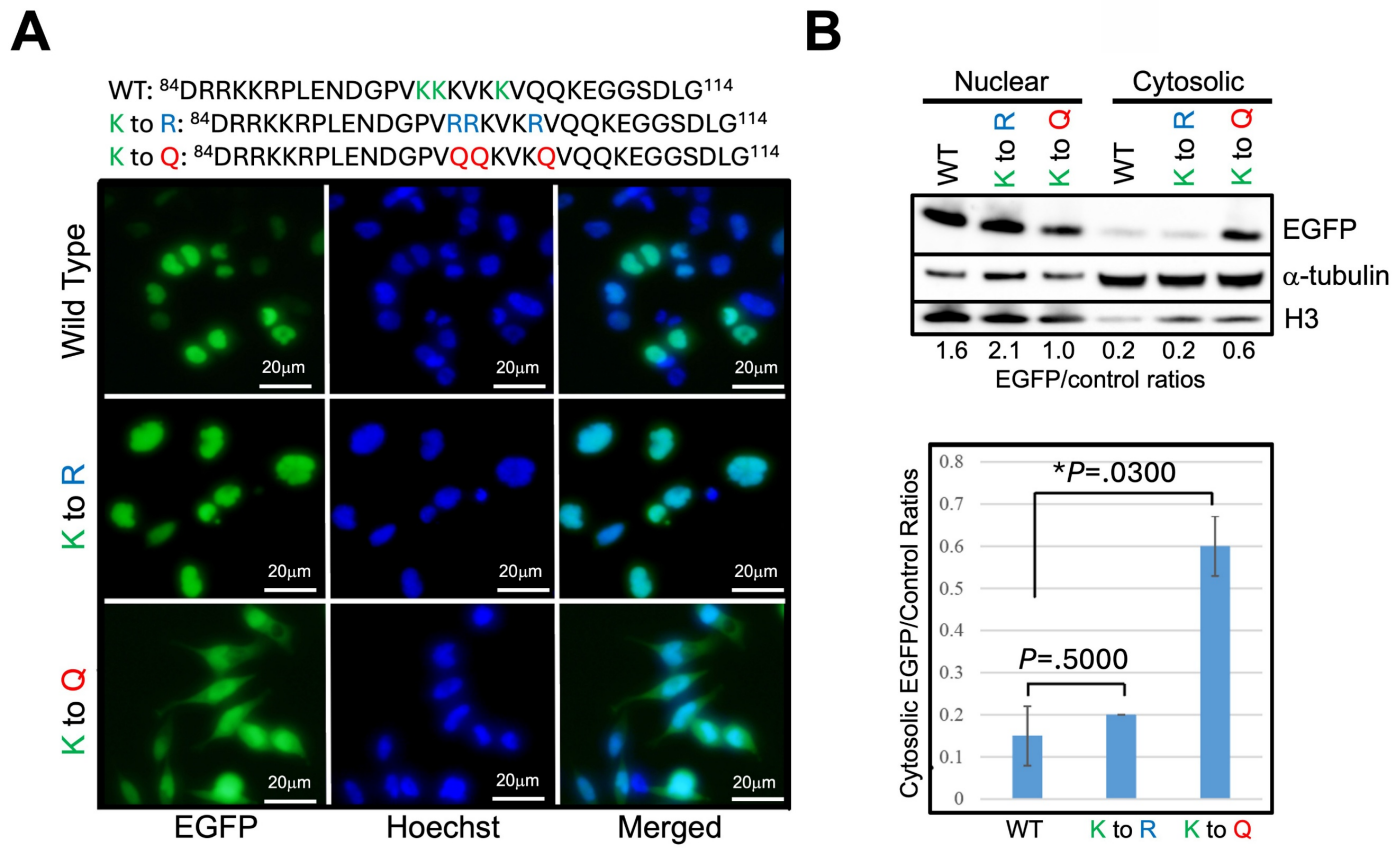
** Acetylation of lysine residues specifically within MSH3's nuclear localization signal help control cytosolic localization.** (***A***) We utilized the reporter construct MSH3-NLS-EGFP 23 with wild type (WT) and adjusted sequences at the three NLS lysine residues as shown (blue and red letters) and transfected into SW480^Δ27/Δ27^ cells. Shown are location of the construct via immunofluorescence microscopy in absence of any treatment. Note that the K to Q (lysine to glutamine) construct, simulating acetylation, shows decreased nuclear with increased cytosolic presence, whereas the K to R (lysine to arginine) construct that simulates deacetylation was completely nuclear. (***B***) (*Upper panel*) Nuclear-cytosolic fractionation and Western blot of cell proteins demonstrate increased cytosolic fraction of the K to Q construct but not that of the K to R construct. (*Lower panel*) Relative protein quantification of the MSH3-NLS-EGFP construct cytosolic fraction showing significant cytoplasmic increase (retention) with the K to Q construct. Histone H3 and α-tubulin were used as nuclear and cytosolic protein controls, respectively. EGFP=enhanced green fluorescent protein.

The effects of TSA and Garcinol are global in their actions. The observation that Δ27bpMSH3's increased cytosolic presence is associated with acetylation of the protein led us to hypothesize that the lysine residues intrinsic to MSH3's NLS might be the critical ones determining subcellular localization. We focused on the second cluster of lysine residues of the bipartite NLS as our previous data indicated that specific cluster was more critical than the first cluster of K/R residues for MSH3's nuclear import function 22. We utilized the reporter construct MSH3-NLS-EGFP 22 and modified the three NLS lysine residues, K^98^, K^99^, and K^103^, that can be acetylated. After transfection of the construct into SW480^Δ27/Δ27^ cells, we employed immunofluorescent microscopy as well as nuclear-cytosolic fractionation and Western blotting to assess subcellular location of EGFP. The construct's lysine residues K^98^, K^99^, and K^103^ were substituted to (a) arginine (R) to mimic un-acetylated lysine residues, (b) glutamine (Q) to mimic acetylated lysine residues, and (c) alanine (A) as a way to completely change the nature of the amino acid. Substitution of each individual NLS lysine residue (e.g. K^98^ alone, K^99^ alone, and K^103^ alone) to another amino acid showed no observable change in the subcellular localization of MSH3-NLS-EGFP (*data not shown*). We then modified the three NLS lysine residues (K^98^, K^99^, and K^103^) as a group, changing all three lysine residues simultaneously to the same substitute. Substituting the three NLS lysine residues to arginine (K to R) solidified the construct's nuclear location (**Figure 3**); substituting the three NLS lysine residues to glutamine (K to Q) moved a significant fraction of the construct from the nucleus to the cytosol (**Figure 3**). These results indicate that unacetylated NLS lysine residues (K^98^, K^99^, and K^103^) are associated with MSH3's nuclear localization, and acetylation of the same residues associate with MSH3's cytosolic translocation. We note that substituting the three NLS lysine residues to alanine (K to A) caused the signal to be cytosolic due to complete absence of nuclear import function with inability to acetylate (or deacetylate) this neutral amino acid (**Supplementary Figure 2**). Overall, acetylation appears to be the key post-translational modification of MSH3's NLS for cytosolic translocation after IL-6, with deacetylation of the same NLS lysine residues important for nuclear import.

### MSH3 binds to the cytosolic enzymatic protein histone deacetylase 6 (HDAC6) after IL-6 treatment

**Figure 4:**
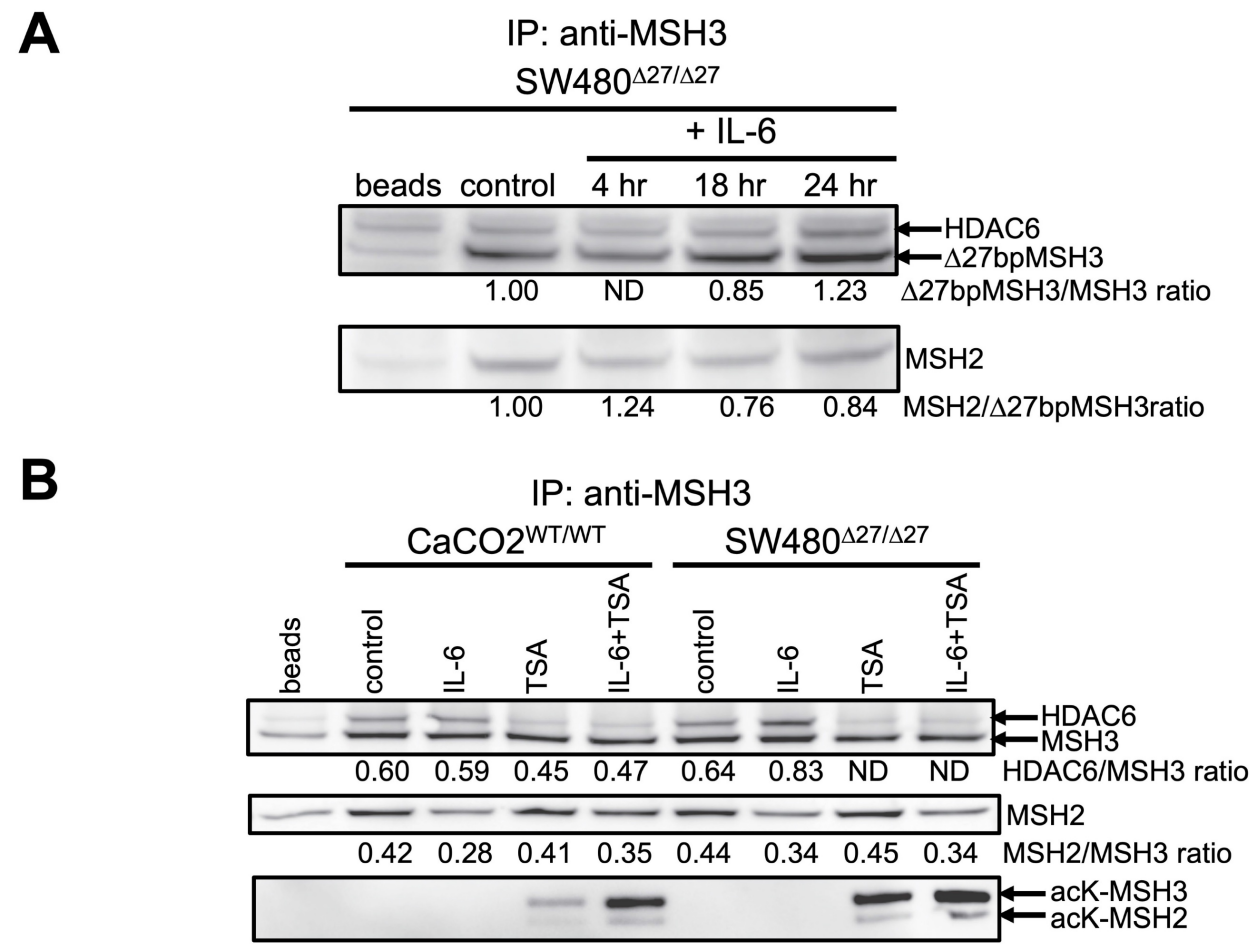
** Δ27bpMSH3 binds to HDAC6 in the cytosol after IL-6 treatment and is blocked by the deacetylation inhibitor trichostatin A (TSA).** (***A***) Immunoprecipitation (IP) with anti-MSH3 pulls down HDAC6 from whole cell lysates from SW480^Δ27/Δ27^ cells at 24 hours after IL-6 treatment, simultaneously with decreased amounts of MSH2. (***B***) Immunoprecipitation (IP) with anti-MSH3 comparing HDAC6 binding in the whole cell lysates of CaCo2^WT/WT^ and SW480^Δ27/Δ27^ cells 24 hours after IL-6 and/or TSA treatment. IL-6 treatment showed no significant interaction between MSH3 and HDAC6 in CaCo2^WT/WT^ cells likely due to less cytosolic translocation of WT MSH3 but showed simultaneous decreased interaction with MSH2. With TSA, MSH3 in CaCo2^WT/WT^ cells showed acetylation at lysine residues, and combined treatment with IL-6 and TSA significantly increased acetylation of MSH3 (and MSH2). IL-6 increased the interaction between Δ27bpMSH3 and HDAC6 in SW480^Δ27/Δ27^ cells that was completely blocked by TSA while simultaneously showing detectable levels of acetylated MSH3 and acetylated MSH2. acK=acetylated K (lysine) residues; ND=not detectable; HDAC6=histone deacetylase 6.

Acetylation and de-acetylation of protein requires interaction with unique proteins capable of that specific enzymatic process. We hypothesized that with our identification of acetylated and de-acetylated states of MSH3 that determine its subcellular location that MSH3 must interact with proteins capable of acetylation and de-acetylation. In examining the literature, histone deacetylase 6 (HDAC6) has been shown to de-acetylate MSH2 and MLH1 obtained from nuclear extracts 26,27, despite HDAC6 residing principally in the cytoplasm and carries a cytosolic signal, which we verified (**Supplementary Figure 1**) 28. Given that when MSH3 is in its acetylated state it is in the cytosol, cytosolic HDAC6 is a likely candidate to de-acetylate MSH3, particularly since it has been shown to interact with other MMR proteins. To assess this possibility, we first performed immunoprecipitation followed by Western blotting of whole cell lysates for Δ27bpMSH3 obtained from SW480^Δ27/Δ27^ cells, since Δ27bpMSH3 is readily translocated in these cells via IL-6 signaling 22. As shown in **Figure 4A**, HDAC6 co-precipitated with Δ27bpMSH3 by 24 hours after IL-6 treatment while simultaneously reducing interaction with its heterodimer partner MSH2, suggestive of a protein swap that may allow MSH3 to maintain its stability. We then performed immunoprecipitation followed by Western blotting of not only the whole cell lysates for Δ27bpMSH3 but also that of MSH3 obtained from CaCo2^WT/WT^ cells to assess effects with treatment with the de-acetylation inhibitor TSA. MSH3, as opposed to Δ27bpMSH3, showed no significant interaction with HDAC6 after IL-6 treatment over controls, likely due to less cytosolic translocation of MSH3 versus Δ27bpMSH3 (**Figure 4B**). However, IL-6 treatment clearly caused reduced interaction between MSH2 with both MSH3 and Δ27bpMSH3, suggestive of both MSH3 isoforms shifting interaction to another protein (**Figure 4B**). Treatment with the de-acetylation inhibitor TSA alone caused increased acetylation of both MSH3 and Δ27bpMSH3 while simultaneously reducing any nascent HDAC6 interaction and completely abolishing any HDAC6-Δ27bpMSH3 interaction; combined treatment by IL-6 and TSA accentuated the acetylation status of both MSH3 and Δ27bpMSH3 without any changes in HDAC6 interaction compared to TSA treatment alone but showed reduced HDAC6 interaction compared to IL-6 alone (**Figure 4B**). HDAC6 remained in the cytosol with all treatments, and all other MMR proteins (MLH1, MSH2, MSH6) stayed in the nucleus (**Supplementary Figure 1**). Note that treatment with TSA also increased the acetylation state of MSH2, consistent with prior reports 26. Overall, polymorphic MSH3 after IL-6 treatment (which induces its acetylated state and cytosolic translocation) binds with HDAC6, which may help stabilize MSH3 after separation from MSH2. The MSH3-HDAC6 interaction presumably prepares MSH3 to become de-acetylated at its NLS and return to the nucleus; Δ27bpMSH3, even with potential de-acetylation by HDAC6 may have difficulty returning to the nucleus due to structural displacement of the NLS for nuclear pore import 22.

## Discussion

MSH3, part of the MutSβ DNA MMR complex, plays an important role for repair of di-nucleotide or longer microsatellite IDLs that occur after DNA synthesis (termed EMAST) 3,14. Absence of MSH3's function, microsatellite repeat sequences frameshift with a bias towards deletion events 11. Loss of MSH3 function may prime cells for further genomic variation and is associated with metastasis and poor outcome 3,14-17,29. Indeed, biallelic germline MSH3 mutations cause somatic dinucleotide or greater frameshifts within the *APC* gene to drive adenomatous oligopolyposis development in this syndrome 18. Sporadic EMAST-positive CRC patients have increased development of metastases that may favor specific genomic events contributing to survival 30, and EMAST accumulates with advancing histological progression through dysplasia and cancer in ulcerative colitis (UC) 23. In both sporadic EMAST-positive CRCs and UC tissues, MSH3 loss-of-function occurs after IL-6 signaling triggers a nuclear-to-cytosolic shift, rendering MutSβ inactive in the nucleus 20-23. Understanding how MSH3 translocates from the nucleus to the cytosol through its post-translational modifications is a key step in potentially mitigating its shift through any future intervention. Here, we examined for IL-6-induced post-translational modifications associated with MSH3 cytosolic translocation. We identified that MSH3 becomes serine then tyrosine phosphorylated with little threonine phosphorylation, and that MSH3 becomes acetylated. The post-translational modification that controls its subcellular localization is acetylation, particularly that of the lysine residues K^98^, K^99^, and K^103^ that are within MSH3's NLS. With post-IL-6-triggered MSH3 acetylation and cytosolic translocation, MSH3 interacts with HDAC6 presumably to de-acetylate MSH3 in preparation to return to the nucleus. All of these findings are novel and contributes to our understanding on how MSH3 is regulated in the cell in response to inflammation.

*MSH3* is polymorphic in its exon 1 sequences, particularly proximate to its mapped NLS 22. The MSH3 polymorph Δ27bpMSH3 modifies MSH3's tertiary structure such that the NLS is less accessible to engage with nuclear pore proteins for nuclear import 22. As a result, Δ27bpMSH3, when triggered to translocate under IL-6 or oxidative stress conditions, becomes sequestered in the cytosol 22. In our current study, we took advantage of Δ27bpMSH3's easy translocation ability for post-translational modifications as a more extreme example of the wild type protein. However, we show that MSH3 undergoes the same post-translational modifications after IL-6 that appear more subtly than Δ27bpMSH3 in some experiments. In any event, we believe our observations for Δ27bpMSH3 are the same that occurs for MSH3.

MSH3 becomes phosphorylated with IL-6 signaling. At present, we do not know the exact functional consequence of the serine then tyrosine phosphorylation, but it appears not directly specific to its nuclear-cytosolic translocation ability like acetylation. One possibility is that phosphorylation allows MSH3 conformational changes during dissociation and association events with protein partners and may have a supporting role in the protein's intracellular translocation.

MSH3's ability to translocate from the nucleus to cytosol with IL-6 signaling appears to be regulated by acetylation. We demonstrate that IL-6 triggers MSH3 to become acetylated, and inhibition of de-acetylation through pharmacological intervention increases the cytosolic presence of Δ27bpMSH3. We demonstrate with the use of a construct that acetylation of three lysine residues (K^98^, K^99^, K^103^) within MSH3's NLS controls cytosolic shift, and through manipulation of the construct to simulate the unacetylated state and acetylated state directed the nuclear and cytosolic location of MSH3. Our prior mapping of NLS has enabled us to examine how MSH3's NLS works. We have since identified polymorphisms that affect the NLS and now have identified acetylation within the NLS as regulators of MSH3's subcellular localization. The NLS lysine residues could be targeted to become de-acetylated and keep MSH3 in the nucleus. Because Δ27bpMSH3 also has structural modification that makes the NLS less accessible, de-acetylation of Δ27bpMSH3 as a strategy to repatriate it to the nucleus may not work due to that structural inaccessibility of NLS.

With identifying acetylation of lysine residues within MSH3 as a key control for nuclear-cytosolic translocation, reversing acetylation is a predictable way for MSH3 to return to the nucleus. We identified HDAC6 as a protein that binds acetylated MSH3. There are likely two functions of MSH3 binding to HDAC6. The first is to bind to and de-acetylate MSH3, reversing the acetylated state, and conferring the ability of MSH3 to return to the nucleus. The second may be one of MSH3 protein stability. We show that with IL-6 signaling, acetylation occurs simultaneously with nuclear-cytosolic shift, and binding to HDAC6 simultaneously with decrease in MSH3-MSH2 interaction. It is presumed that the MSH3-HDAC6 interaction, once MSH3 is de-acetylated, would return to the nucleus and be again bound to MSH2 for stability. It is possible that for Δ27bpMSH3, which with de-acetylation is largely unable to return to the nucleus due to NLS inaccessibility, can bind to other proteins aside from HDAC6 that contributes to its stability when not bound to MSH2. The cytosolic HDAC6-MSH3 interaction we identified is in addition to a predominantly nuclear located HDAC3-MSH3 interaction described for regulation of triplet repeat expansions in neurons, with predominance in de-acetylating lysine residues K^122^, K^123^ and K^99^ proximate and in MSH3's NLS, and lesser ability to de-acetylate K^98^ and K^103^ within MSH3's NLS 31. It is possible that both HDAC3 and HDAC6 have roles in their respective compartments that affect the MSH3-MSH2 interaction as well as intracellular compartmentalization and protein stability. Furthermore, recently we identified NEMO/IKKγ as a binding and protein stability partner for MSH3 after double strand breaks occur 24.

In conclusion, polymorphic MSH3 undergoes serine/tyrosine phosphorylation and NLS acetylation upon IL-6 signaling for its nuclear-cytosolic shift, with NLS acetylation controlling the shift capability. MSH3 binds HDAC6 in the cytosol with may contribute to anticipated deacetylation as well as to MSH3 protein stability along with NEMO/IKKγ 24,32 when not bound to nuclear MSH2. These changes might be targeted to regulate MSH3's intracellular location. Clinically, for colorectal cancer patients, reversing the inflammation-induced MSH3 displacement might reduce its association with advanced cancer stage and aggressive biological behavior and has the potential to improve survival.

## Materials and Methods

### Cell culture and reagents

All cells were obtained from ATCC. CaCo2^WT/WT^ cells were cultured in DMEM media (Invitrogen), while SW480^Δ27/Δ27^ cells were cultured in IMDM (Invitrogen), with both cell cultures supplemented with 10% FBS and 1X pen-strip (Invitrogen). Both cell lines were chosen specifically for their MSH3 polymorphic genotype to compare. Human recombinant IL-6 was purchased from R&D System (Minneapolis, MN). Polyclonal rabbit anti-MSH3 antiserum was a kind gift from Dr. Giancarlo Marra (University of Zurich, Zurich, Switzerland) 33. Monoclonal mouse anti-MLH1, anti-MSH2, anti-MSH6, and anti-MSH3 antibodies were purchased from BD Biosciences (San Jose, CA). Monoclonal rabbit anti-MSH2, monoclonal rabbit anti-MSH3, polyclonal rabbit anti-MSH3 C-terminal, anti-phospho-serine, anti-phospho-tyrosine, and anti-HDAC6 antibodies were ordered from Abcam (Cambridge, United Kingdom). Anti-phospho-threonine was from Cell Signaling (Danvers, MA). Rabbit TrueBlot WB analysis kit was from Rockland Assays & Antibodies (Gilbertsville PA) for the detection of the protein of interest after IP experiments. Garcinol was purchased from Fisher Scientific (CAS 78824-30-3) and trichostatin A (TSA) was purchased from Sigma-Aldrich (T8552).

### Immunoprecipitation (IP) and western-blotting (WB)

Cells were split to encourage active growth and incubated at 37 °C/5% CO_2_ overnight. Cells were subjected to serum starvation for 18 hours, followed by treatment of 20 ng/ml IL-6 for 18 hours. The next day, cells were treated with another dose of IL-6 for 4 hours. Cells were then collected using cell scrappers and counted. An equal number of cells was used to prepare cell lysates using Native Sample Prep kit (Invitrogen). Anti-rabbit IgG beads (50 μl per reaction; Rockland Antibody & Assays) were washed with PBS, equilibrated with cell lysis buffer, and incubated with cell lysate and the antibody at 4 °C for 2-3 hr. Cell lysates were removed, and the beads were washed with cold wash buffer (0.2% TW-20 in PBS) 4 times, 5 minutes each wash. Beads were heated with 50 μl of 2X SDS-PAGE sample buffer at 100 °C for 10 minutes, followed by centrifugation at 10,000x g for 5 minutes. The supernatants were collected for WB analysis as described 20 using the Rabbit TrueBlot WB kit to eliminate the appearance of the bands corresponding to rabbit antibodies. The intensity of the bands was quantitated to calculate the relevant ratios as described 21. All experiments were performed at least twice. Student 2-tailed t-tests were conducted for the statistical analyses.

When cytosolic fractions were used for the IP experiments, beads were rinsed in 1X Buffer A before incubating with cytosolic fractions and the antibody.

### Nuclear and cytosolic protein fractionation

The fractionation was performed using the Cell Fractionation Kit (Abcam) following instructions with minor modifications. We combined mitochondria and nuclear fractions to calculate the fold change of the percentage of cytosolic MSH3 upon treatments. Briefly, cells from one 10-cm dish were collected and resuspended in 5 ml of 1X Buffer A to wash and count cells. Cells were centrifuged at 300X g and then resuspended in 1X Buffer A to adjust cell density to 6.6 million cells per ml. Fifty μl of cells (330,000 cells) was used for each reaction. Fifty μl of freshly prepared Buffer B with proteinase inhibitor cocktail (Cell Signaling) was added into each tube, and cells were incubated on a rotator at room temperature for 7 min, followed by centrifugation at 5,000X g at 4°C for 1 min. The supernatant was collected, separated, and re-centrifuged at 10,000X g at 4 °C for 1 min. The supernatant was recollected, which was the cytosolic fraction. The pellets from both centrifugations were pooled and resuspended in 50 μl of 1X Buffer A, to which 50 μl of 1X Buffer A was added and centrifuged immediately at 5,000X g at 4 °C for 1 min to wash the pellets. The pellets were resuspended in 100 μl of 1X Buffer A with proteinase inhibitor cocktail, individually. Twenty-five μl of 5X SDS-PAGE sample buffer was added into each cytosolic and/or nuclear fraction and heated at 60 °C for 10 min. Equal volume of cytosolic and nuclear proteins was used for the subsequent WB analysis.

To prepare cytosolic fractions for immunoprecipitation experiments, the fractionation volume was scaled up 10 times to 0.5 ml, and all reagents used were scaled up 10 times as well.

### Immunofluorescent microscopy (IFM)

Cells were seated onto the 4-well chamber slides (40,000 cells/well) and incubated at 37 °C/5% CO_2_ overnight. Upon completion of IL-6 or H_2_O_2_ treatment, cells were fixed with iced-cold acetone for 5 minutes, air dried, and stored at 4 °C until staining. Staining has been described previously 21,22.

### Generation of modified MSH3-NLS-EGFP expression constructs

PCR mutagenesis was performed to generate the various constructs for the studies investigating the effects of NLS substitutions and MSH3 shuttling. An expression construct carrying the NLS of MSH3 cDNA tagged with EGFP tag 22 was used as a template for PCR mutagenesis into various lysine residue substitutions. Each construct was subjected to DNA sequencing to confirm the desired mutations.

After cells were seeded onto 4-well chamber slides and incubated at 37 °C/5% CO_2_ overnight, expression constructs were electroporated into cells, separately, as previously described 21. The next day, cells were subjected to serum starvation for 18 hours, before treatment with IL-6. Cells were fixed with iced-cold acetone for 5 minutes and air-dried. IFM was performed as described previously 20-22. The percentages of the MSH3-expressing cells exhibiting significant cytosolic MSH3 staining (losing appearance of nuclear enrichment) were calculated. Two-tailed student t-tests were performed for statistical analyses.

## Supplementary Material

Supplementary figures.

## Data Availability

The authors confirm that the data supporting the findings of this study are available in the article and/or its supplementary materials. The authors agree to make data and materials supporting the results or analyses presented in the paper available upon reasonable request.

## Abbreviations

MMR: DNA mismatch repair
MSI: microsatellite instability
IL-6: interleukin-6
NLS: nuclear localization signal
IFM: immunofluorescence microscopy
WB: Western blotting
IP: immunoprecipitation
RT-PCR: reverse transcriptase polymerase chain reaction
ROS: reactive oxygen species
WT: wild type
TSA: Trichostatin A
HDAC: histone deacetylase
