# Supplementary material for: Inflammation Drives Phosphorylation and Acetylation of MutS Homolog 3 and Interaction with Cytosolic HDAC6
Source: J Cancer. 2026 Mar 25;17(4):730–9. doi: 10.7150/jca.131728 (PMC13104720; doi:10.7150/jca.131728)

Supplementary Figures and Figure Legends

Supplementary Figure 1. Nuclear-cytosolic protein distribution after treatment with IL-6, Garcinol, and/or trichostatin A (TSA). The distribution pattern of Δ27bpMSH3 changed upon all single and combined treatments, while that of WT MSH3 was only altered significantly to the cytosol upon treatment involving IL-6. No treatment markedly changed distribution of MLH1, MSH2, MSH6, and/or HDAC6. N=nuclear, C=cytosol, H3=histone H3

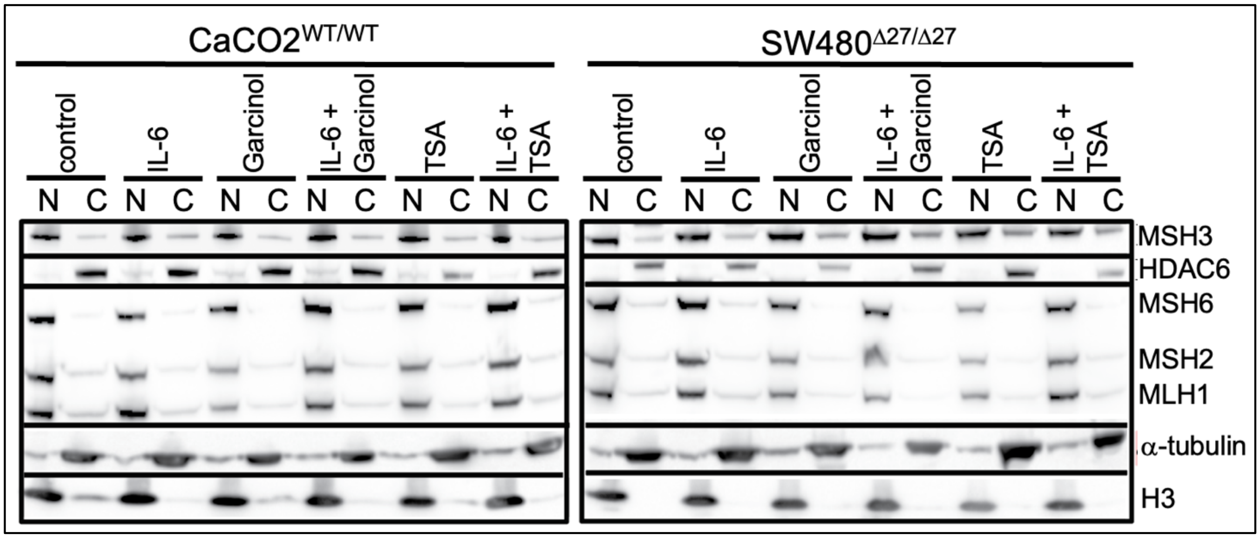

Supplementary Figure 2. SW480$^{\Delta27/\Delta27}$ cells transfected with the K to A (lysine to alanine) MSH3-NLS-EGFP construct exhibited significant cytosolic signal. Changing all three lysine residues to alanine severely impaired the nuclear localization function of the MSH3 NLS. EGFP=enhanced green fluorescent protein

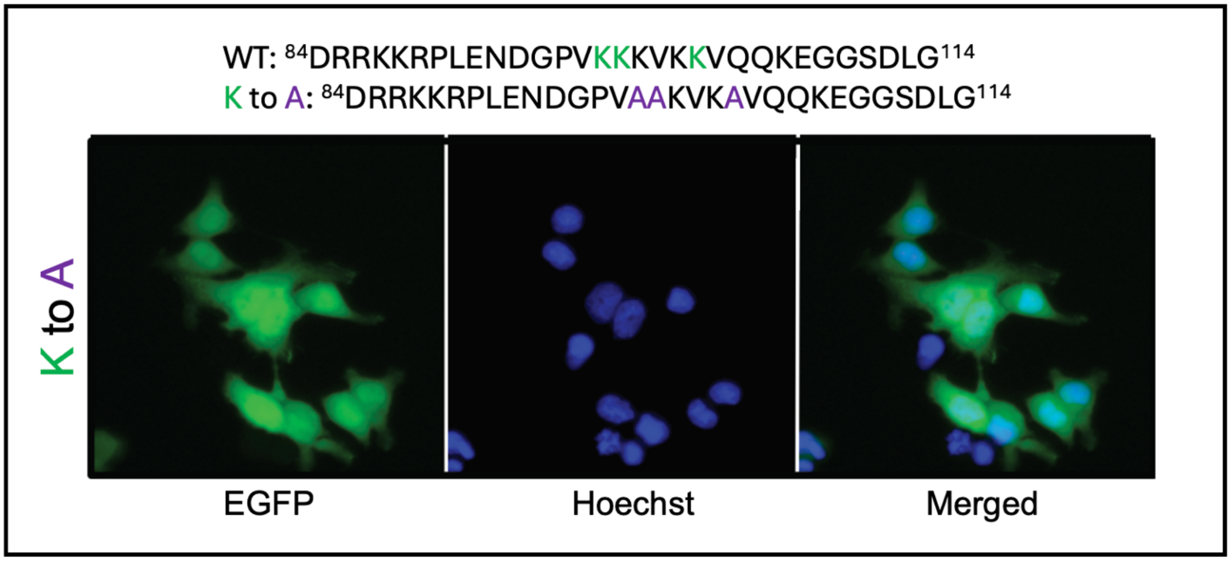

Supplement: Supplementary file 1 — Supplementary figures. [file jcav17p0730s1.pdf]
